# Clinical research updates

**DOI:** 10.1111/camh.12742

**Published:** 2025-01-22

**Authors:** Marinos Kyriakopoulos, Ifigenia Metaxa, Caitriona Cotter, Isidora Fili

**Affiliations:** ^1^ Department of Psychiatry, Eginition Hospital, National and Kapodistrian University of Athens Athens Greece; ^2^ Department of Child and Adolescent Psychiatry, Institute of Psychiatry Psychology and Neuroscience King's College London London UK; ^3^ South London and Maudsley NHS Foundation Trust London UK

## The experience of depression by adolescents

Ifigenia Metaxa

National and Kapodistrian University of Athens

Depression constitutes a serious burden to adolescents all around the world. Understanding the perspective of those living with the disorder may assist in better identification and development of effective treatment approaches.

Viduani et al. (2024) conducted a systematic review of qualitative studies exploring the subjective experience of depression in young people (age range 10–24 years). A total of 39 studies were included, representing the experiences of 884 adolescents with depression from 16 different countries. The authors identified 47 features of depression. Among the 10 most cited features, 5 were part of DSM/ICD diagnostic criteria, namely, sadness, worthlessness, loss of energy, hopelessness and motivational anhedonia. The remaining features listed were social withdrawal, loneliness and anger together with stress and frustration or feelings of failure.

Following a meta‐synthesis approach, it was found that lived experience of adolescent depression can be understood through the lens of 3 overarching themes. The first theme is related to the meaning that adolescents give to depression, including the impact on their life, relationship difficulties, self‐blame, guilt, fear of isolation and mood changes. Most adolescents reported using self‐reliant strategies, such as distraction or expressing negative emotions through art or religion, but some used alcohol, drugs, self‐harm or other risky behaviours to manage their feelings. The second theme involved cultural and societal aspects of depression. Depression was found to be related to factors including cultural norms, social conditions, life events, family relations and peer group pressure. Some adolescents reported being victims of sexual abuse. Across contexts, interpretations and expectations related to depression differ between boys and girls and seem to also be affected by race and ethnicity. Depression was also found to have a long‐term impact on education, while fatigue and low motivation may negatively affect academic progress. Social relationships were also affected, with adolescents reporting an influence on communication and social withdrawal. In these situations, they reported thoughts of dying and suicide. The third theme highlights adolescents' efforts to access care and support networks. Different factors were identified as barriers to help‐seeking. First, adolescents avoid self‐disclosure because of fear of stigma and negative reactions from parents and peers. They also mentioned feeling let down by the systems, leading to a lack of hope in sources of help, while limited resources were also an issue. In addition, adolescents reported distrust in professionals and perception of inefficacy of treatments, both pharmacological and psychotherapeutic. They also expressed a sense of not being heard or explained about the purpose and utility of treatments used (mostly medication) and expressed the need of having providers as allies, not authoritarian leaders.

In both feature‐driven and qualitative approaches, the findings suggest that focusing solely on DSM/ICD criteria might only partially capture the experience of depression among youth. The generated themes further emphasize the relevance of impaired social function in depression during adolescence, which is consistent with previous literature.

**Reference**

Viduani, A., Arenas, D.L., Benetti, S., Wahid, S.S., Kohrt, B.A., & Kieling, C. (2024). Systematic Review and Meta‐Synthesis: How Is Depression Experienced by Adolescents? A Synthesis of the Qualitative Literature. *Journal of the American Academy of Child and Adolescent Psychiatry*, 63(10), 970–990.

## A thematic analysis of #camhs on TikTok

Caitriona Cotter

South London and Maudsley NHS Foundation Trust

Understanding the barriers young people face when seeking mental health help is essential. Young people are increasingly turning to social media to explore and share content related to mental health, with TikTok being a popular platform. Exploring TikTok videos provides an opportunity to gain first‐hand, candid reflections from young people about their experiences in Child and Adolescent Mental Health Services (CAMHS), which differs from traditional feedback channels.

The study by Foster et al. (2024) used an inductive framework thematic analysis to examine 100 TikTok videos tagged with #camhs posted between 2019 and 2021. These videos averaging 17 s, had a range of engagement metrics that showed high levels of interest among viewers. A participatory approach was used to ensure the young person's voice was not ‘lost’, with five co‐researchers, 15–17 years old, recruited. Data analysis was a five‐stage framework with codes generated collaboratively with co‐researchers, initially from a subset of videos, before being refined and organized into a codebook for systematic application across the full data set. Through an iterative process of reviewing and connecting codes, four primary themes were collaboratively created with the co‐researchers: (a) CAMHS can be frustrating and unhelpful, but sometimes life‐saving, (b) Young people can feel their distress is invalidated by CAMHS, (c) CAMHS makes young people feel responsible for their distress and (d) Young people may not feel CAMHS professionals are trustworthy.

Findings indicate widespread dissatisfaction with CAMHS, with young people conveying the interventions as ineffective and invalidating, done ‘to’ them, rather than collaboratively ‘with’ them. Examples cited clinicians appearing to respond to severe distress by offering ‘basic’ advice, such as take a bath or have a cup of tea; as well as seeming to withhold a formal diagnosis, leaving many young people confused and dismissed that they do not have depression, but low mood, which further reinforced a power imbalance. Systemic issues, including long waiting times and a sense that clinicians were more focussed on administratively managing risk over immediate mental health needs, added to young people's negative view of CAMHS. However, a minority of videos conveyed positive experiences, highlighting instances where young people felt CAMHS clinicians listened to them and actively involved them in their care.

This study suggests that TikTok appears to offer a space for young people to voice their experiences and seek validation from peers, perhaps filling a gap that CAMHS have not met. It is pertinent these findings are considered in the context in which they were posted, during the COVID pandemic when CAMHS were likely to be experiencing significant organisational challenges. Furthermore, to consider whether the purpose of these posts is purely informational or also to elicit an emotional response. This study suggests practical considerations for CAMHS clinicians to enhance therapeutic engagement including validating a young person's distress; clarifying the purpose of coping strategies and how they work physiologically; discussing confidentiality transparently; and more actively engaging young people in their care decisions. Further research could explore how professionals can communicate effectively with a young person to honour their autonomy while also considering the risks and benefits of receiving a diagnosis or not.

Strengths of the study include its innovative use of TikTok to access young people's perspectives of their CAMHS experiences and the involvement of young people as co‐researchers in data analysis. Limitations include a potential lack of representativeness, as videos may not reflect the experiences of all CAMHS users. Most videos in the sample appear to have been curated by white, females, yet previous research indicates help‐seeking behaviour is significantly lower in males and people from ethnic minority groups, indicating an area for further study.

**Reference**

Foster, M., Frith, H., & John, M. (2024). ‘I'm still su!c!dal when you're done with the paperwork’: an inductive framework thematic analysis of #camhs on TikTok. *Journal of Child Psychology and Psychiatry*, 65, 1258–1269.

## Childhood and adolescence outcomes in offspring to parents with bipolar disorder

Isidora Fili

National and Kapodistrian University of Athens

Bipolar disorder is a highly heritable condition associated with significant morbidity. Children of parents with bipolar disorder are also at risk of multiple other psychiatric conditions, somatic illnesses and accidents.

Takami Lageborn et al. (2024) explored, through a cohort study in Sweden, the outcomes in offspring to parents with bipolar disorder. Swedish population registers were linked to compare offspring having (*N* = 24,788) and not having (*N* = 247,880) a parent with bipolar disorder with respect to psychiatric diagnoses and psychotropic medication, birth‐related and somatic conditions, social outcomes, accidents and mortality. Individuals were followed until the age of 18. The influence of lifetime parental psychiatric comorbidity, bipolar disorder subtype and sex was also evaluated.

The study identified that children of a parent with bipolar disorder had 2–3 times increased risk for all psychiatric diagnoses, the highest being for bipolar disorder (11 times higher). Having two parents diagnosed with bipolar disorder showed the highest risk for bipolar disorder in offspring (15 times higher). Having two parents with bipolar disorder, having a parent with bipolar disorder type 2 and having a mother with bipolar disorder showed an increased risk of receiving any psychiatric diagnosis and being prescribed ADHD medication and melatonin. Bipolar disorder in parents was also associated with an increased relative risk of perinatal period conditions, congenital malformations and several types of somatic health conditions in children. In addition, an association was identified between parental bipolar disorder and asthma in offspring, especially in offspring of mothers with the disorder. In relation to neurological conditions, parental bipolar disorder was associated with an increased risk of epilepsy, migraine and narcolepsy in offspring. Finally, higher risk of low school grades, accidents and suicide attempts were linked with parental, especially maternal, bipolar disorder.

This study had a few limitations. In this cohort, there had been limited data up until 2013, and data on prescribed medications started in 2005. In addition, the coverage of bipolar disorder quality register was incomplete in about one third of patients in Sweden. Furthermore, parental education in this study was used as a proxy marker for socioeconomic status. Finally, the conclusions may not be transferable to other countries as psychiatric diagnostic practices may differ across the world. However, this is the largest and most extensive study to date investigating the outcomes of offspring born to parents with bipolar disorder. The findings corroborate previous evidence underscoring that offspring of parents with bipolar disorder face increased risks across a range of psychiatric diagnoses, somatic conditions and adverse social outcomes. Clinicians and policymakers should be aware that children with such a family history may be in greater need of early support and treatment.

**Reference**

Takami Lageborn, C., Zhou, M., Boman, M., Sjölander, A., Larsson, H., D'Onofrio, B.M., … & Landén, M. (2024). Childhood and adolescence outcomes in offspring to parents with bipolar disorder: The impact of lifetime parental comorbidity, parental sex, and bipolar subtype. *Journal of Child Psychology and Psychiatry*, 65, 1355–1368.

## Ethical information

No ethical approval was required for these updates.

## Data Availability

Data sharing is not applicable to this article as no new data were created or analyzed in this study.

